# Burrow characteristics of the mud shrimp *Austinogebia edulis*, an ecological engineer causing sediment modification of a tidal flat

**DOI:** 10.1371/journal.pone.0187647

**Published:** 2017-12-13

**Authors:** Shagnika Das, Li-Chun Tseng, Lan Wang, Jiang-Shiou Hwang

**Affiliations:** 1 Institute of Marine Biology, National Taiwan Ocean University, Keelung, Taiwan; 2 School of Life Sciences, Shanxi University, Taiyuan, China; Zhejiang University College of Life Sciences, CHINA

## Abstract

The mud shrimp *Austinogebia edulis*, being abundant in the intertidal zone of western Taiwan, constructs deep burrows (>1 m). This study highlights the potential of mud shrimps to modify sediment characteristics of the tidal flat by its burrowing behavior. We studied the structure of the burrow wall, compared the difference in the sediment composition of the burrow and the background sediment, and compared the organic content inside the burrow wall. This study was carried out from September 2015 to November 2016 in three areas of the western coast of Taiwan, namely Shengang, Hanbow, and Wangong. The present study found significant differences between burrow wall and the burrow lumen. The diameter of the burrow wall was double as wide as the inner burrow lumen at the opening and gradually increased to 10 times of the burrow lumen at 30 cm depth. The burrow wall of *A*. *edulis* showed low permeability and increased the sheer strength. Statistically, a significant difference was noticed in the comparison between the sediment composition of the burrow wall and the background (*p* < 0.05, Student’s t-test). An accumulation of 3.63 for fine sand (t = -5.22, *p* < 0.001, fine sand) and 9 for clay (t = -25.01, *p* < 0.001, clay) was found in the upper burrow wall of *A*. *edulis*. This indicated that they somehow chose finer particles to build burrows. This will gradually change the sediment distribution—vertically and horizontally. The burrow wall consisted of a 24 times higher organic matter content than one individual of mud shrimp. The burrow may provide organic material as a potential food source. The mud shrimp thus transforms the sediment characteristics as an ecological engineer, which is expected to have a significant ecological impact on the ecosystem.

## Introduction

Mudflats are coastal wetlands that are formed by the sedimentation of mud layers during tidal movements [1]. Generally, these layers are made of sand, silt, or clay. Tidal flats constitute a transition zone between land and sea [2]. Tidal flats are habitats to different kinds of organisms like, benthic burrowers, microalgae, and even bacteria. They are important wetlands where numerous biological activities take place. Many species of crabs, clams, shrimps, fish etc. hide there by creating burrows into the sediment [3]. Among them are mud shrimps that dig complex and deep burrows [4–7]. Some mud shrimp species are known to dig more than 2 meter deep burrows, for which it has been always been difficult to acquire a holistic approach to their behavior [8, 9].

**Fig 1:**
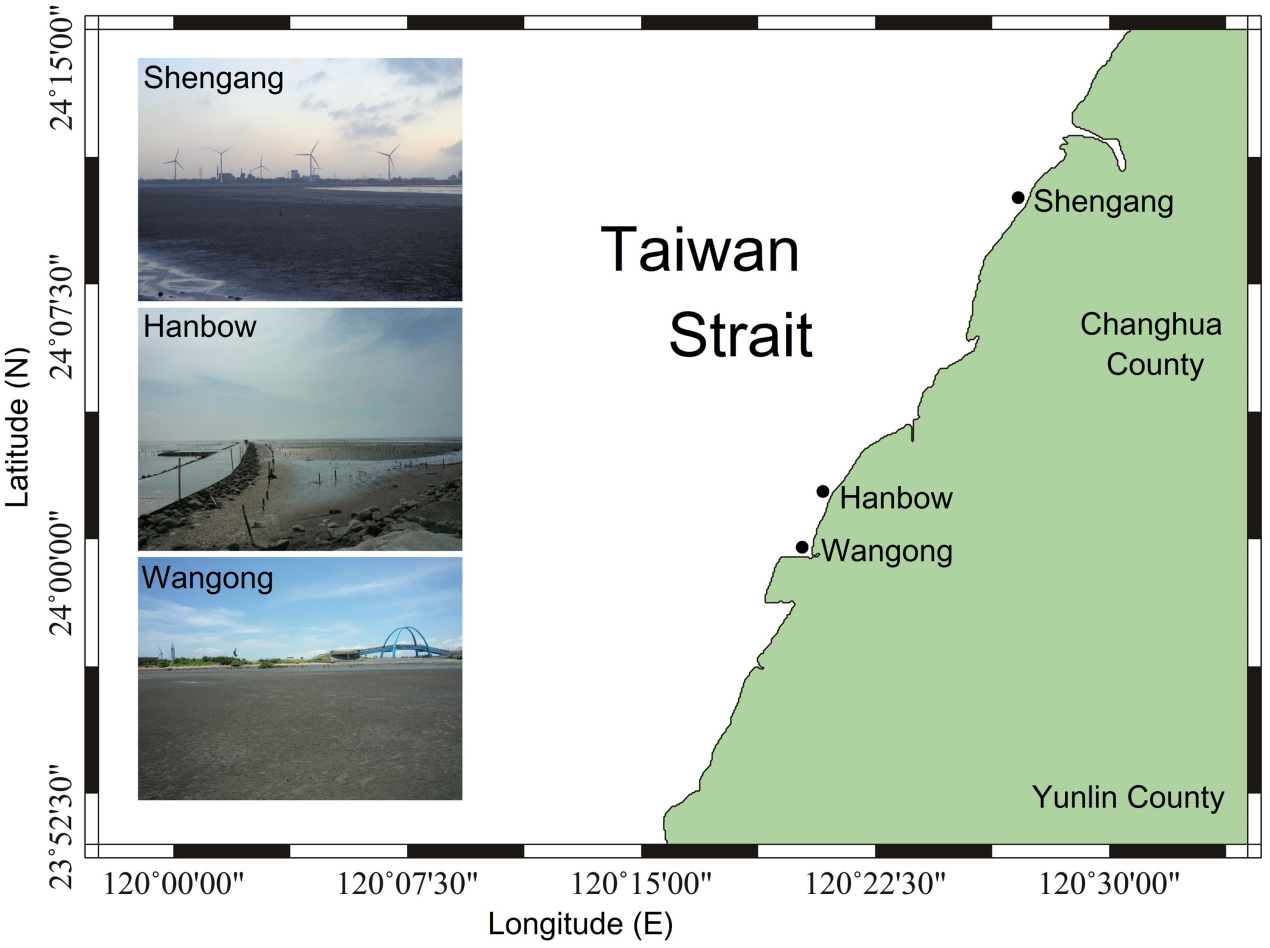
Map of the sampling area in western Taiwan.

In the coastal wetlands of western Taiwan and northern Vietnam, the mud shrimp *Austinogebia edulis* [10] (Fig 1) is abundant and of economic importance as seafood. The species *Upogebia edulis* was revised to *Austinogebia edulis* after the re-classification of upogebiid species into the genus *Austinogebia*. [11]. The locals of western Taiwan catch and consume this shrimp extensively [12] and the ovigerous female shrimps occur only found during the reproductive season and are more expensive than the males or non-ovigerous females. Mud shrimps are cryptic animals that prefer to reside inside their burrows in deeper layers of sediment. Their burrows are only recognizable through small burrow openings [12, 13–16, 17]. With the advent of the resin casting technique [18] the interior morphology of mud shrimp burrows received great attention, which greatly improved the understanding of their burrow structures [8, 19–26]. Reports of resin casting in the inner burrow structure of *A*. *edulis* showed that these are generally Y shaped with an upper U part and a lower shaft [27, 28]. One mud shrimp burrow has usually two openings and the mean distance between them is 21.8 cm [29] and 26.4 cm [27]. A single shrimp generally inhabits it. Studies on the outer morphology of the burrows have not been done as yet.

We observed that the burrow wall composition was different from ambient uninhabited sediments without the burrow. A previous study on the grain size of the sediments from abundant areas of *A*. *edulis* revealed that these were mainly composed of fine silt (0.061mm), but a detailed analysis between burrow and areas without burrows was not made as yet [27]. Therefore, we studied here whether mud shrimps could change the sediment characteristics while constructing their burrows acting as ecological engineers with substantial ecological impact. Previous studies reported the effect of burrowing behavior of mud shrimps on the ecology of tidal flats, mainly examining factors such as the bacterial abundance, change of oxygen and nutrient fluxes, organic content inside the burrow and its potential to change the environment [6–7, 30–35]. The burrow of *A*. *edulis* is more than 1 meter deep and this species is abundant in the wetlands of western Taiwan [27]. Therefore, the influence of this thalassinidean shrimp on the alteration of tidal flats must be substantial and they are expected to affect other benthic animals living in the surroundings. Hence, studies on the composition (grain size analysis) of the mud shrimp burrow wall (MSBW), and its comparison with the sediment from the background (a place without mud shrimp burrows) is timely.

In the laboratory, *A*. *edulis* rejected food offered into their burrow opening such as fish and shrimp meat, planktonic algae, dead copepods, and aquaculture feed of shrimp larvae (Das et al.unpublished data). From this observation, this shrimp is perhaps not a pure filter-feeding animal.

While studies have indicated substantial changes of the environment caused by the mud shrimp, it remains also important to calculate the substantial amount of carbon inside their burrows as a food source [32, 36–37]. Amongst thalassinidean shrimps, Upogebiidae are considered mainly as filter feeders but some representatives also show plasticity for feeding behavior [4,9]. The burrow provides a steady water flow and a stable carbon source in the burrow for the animals living inside [22]. Therefore, an estimation of the organic carbon in the MSBW that might be utilized by the mud shrimp *A*. *edulis* would be worthwhile.

The main objective of this study was to test the hypothesis whether the mud shrimp can change its environment by building a burrow. In addition, we addressed the following ecological issues, to study: (1) the outer morphological structure of a mud shrimps burrow wall and its characteristics; (2) the potential of mud shrimps to modify a tidal environment by selecting and fractionating sediments in the process of burrow building; (3) to measure the organic matter in a MSBW.

## Material and methods

### Study area

For sampling, we chose three sampling areas from north to south, which are tourist attractions in Changhua County (Fig 1). The areas of investigation were: Shengang in the northern part located close to the industrial park, Hanbow in the central, and Wangong in the southern part along the western coast of Taiwan facing the Taiwan Strait (Table 1). Our study was permitted and supported by the Industrial Development Bureau, Ministry of Economic Affairs. This study did not involve specimens; tissue samples or any endangered or protected species. The climate of Taiwan is affected by seasonal monsoons with the air temperature being 12°C in winter and 30°C in summer. Ocean currents in this region are influenced by seasonal monsoons. In summer, the Kuroshio Branch Current and the South China Sea surface water enter the Taiwan Strait from the south. In winter, the China Coastal Current enters the Taiwan Strait from the north [38].

**Table 1:** Sampling period, location and coordinates of experimental specimen collections from different mudflat environments in western Taiwan.

Sampling period	Sampling location (ca.)		
	Local name	Latitude (N°)	Longitude (E°)
September 2015–November 2016	Shengang	24.168094	120.457894
	Hanbow	24.015691	120.349280
	Wangong	23.968126	120.323173

### Sampling strategy

We conducted the field sampling from September 2015 to November 2016. Samples of mud shrimp *A*. *edulis* burrows were collected carefully by using a shovel or small rake or fork. A densely populated area was randomly chosen to be sampled with a shovel; a portion of mud block containing burrows of mud shrimps was scooped out. As the burrows were very distinct from the background sediment in terms of texture, hardness, compactness, and shape, this enabled an easy separation of muddy burrow blocks and loose sandy background sediments. The samples of burrow and background sediment were both collected from above 30 cm depth. We washed the burrows gently to remove loose sediments attached to it. A total of 50 samples from burrow and 50 samples of surrounding sediment were randomly collected from each area. We collected background sediment from areas comprising no mud shrimp burrow as a control to be compared with the burrow sample. In total, about 300 sediment samples were collected to find out the difference in the composition and to measure the ash free dry weight (AFDW, organic matter, carbon content) for three areas. After collection, the samples were placed carefully in separate zip-lock bags and were carried to the laboratory. In the laboratory, the samples were then stored in a -20°C refrigerator until analysis. In addition, for measuring the diameter of the burrow we collected 3 resin casts of the burrow of *A*. *edulis* in November 2015 from Hanbow according to Li. et al, 2008. We measured the diameter of the burrow at every 10 cm depth from the surface to the bottom in all three resin casts.

The other objective was to study the structure of the burrow wall of *A*. *edulis*, which required a different technique. The objective was to acquire the complete burrow shape and to show thick patches of fine sediments accumulated by the mud shrimp during their burrow-building processes. A considerable depth and a wide surface area containing at least a major portion of one burrow with two openings were necessary to collect. Wooden planks were hammered into the sediment from all four sides for taking a mud block of the following size 30 × 30 × 30 cm^3^ that contained a portion of the outer dimension of a burrow. After this, two shovels were inserted from two opposite sides into the bottom of the mud block. This was done at all four sides for easy removal of the mud block from the tidal flat. Then, we wrapped and tied the planks with adhesive tapes repeatedly to make the structure firmer. The wooden box was then lifted up carefully from the mud flat and was carried to the laboratory.

There, after the removal of the wooden planks a weak water stream comparable to the tidal hydrological force was used to remove the loose sediment. Coarse sediment that was not part of the burrow was gently washed away, and the hard burrow structure got gradually exposed. This burrow structure was used to measure the traits of the outer wall and for photo documentation.

### Sediment handling and analysis

We randomly selected 8 samples of burrow and 8 samples of background sediments in total from each sampling area in order to measure the sedimentary composition. Particle size was determined by passing each sample of sediment through a series of sieves. The fraction remaining on each sieve was collected in a pre-weighed 100 ml beaker. A total of 7 mesh sizes (4, 2, 1, 0.5, 0.21, 0.105 and 0.063 mm) were used to pass the samples and gradually separate them into different size groups. After collecting the remaining fraction, the total weight was again noted for each size fraction and was expressed as a percentage of the weight of the original sample.

For measuring the volume of the burrow wall, the portion of one entire burrow collected from 30cm depth was wrapped tightly with Polyethylene wrap film to measure the total volume by using the water displacement method [39]. For measuring the burrow lumen,

$$Volume=\pi \times r^{2}\times length$$ (1)

where, r = radius of the resin casting, which is 2 cm (from the resin cast collected in November 2015), length is the height of the mud block, which is 30cm.

For revealing the variability of sedimentological characteristics of mud shrimp burrows, we used a total of 12 samples from three sampling areas to measure the void ratio (*e*).

e=Vv÷Vs (2)

where V*v* is volume of void (equal to volume of water), V*s* is volume of solid sediment sample (equal to volume of burrow sample).

### Estimation of organic matter of the mud shrimp burrow wall

We weighed a total of 10 adult individuals of mud shrimps (5 male and 5 female, carapace length: 12.22–13.97 mm, 13.39 ± 0.69 mm (mean ± standard deviation), 4 samples of background sediment and 12 samples of the inner surface of the mud shrimp burrow from three sampling areas for wet weight (WW). The dry weight (DW) of the samples was determined by drying in an electric oven at a constant temperature of 60°C for 24 hours. Both WW and DW were measured by analytical microbalance (Type AG 135, Mettler Toledo, Switzerland) and recorded. Dried samples were then placed in an electric oven and combusted at a constant temperature of 500°C for 16 hours to measure the ash weight (AW). For revealing the organic content of the samples, the AFDW was calculated by deducting the AW from the DW.

In order to calculate the organic matter of the MSBW, a definite volume of the burrow sample was required. For this, a volume of 10 cm^3^ burrow sediment from the inner surface was used to measure the AFDW. Hence, the estimation of the AFDW of one whole mud shrimp burrow was following the Eq (3):

AFDWt=IDB×π×LB×AFDWbm÷Vm (3)

Where AFDW*t* is represented by the total AFDW in one whole mud shrimp burrow, IDB is the inner diameter of the burrow, LB is the length of the burrow, AFDW*bm* is the AFDW in the burrow mud, and V*m* is the volume of the burrow mud used to measure the AFDW. In this study, the inner diameter of the burrow was 20 mm from the resin samples collected in November 2015 (20 ± 0.12 mm). The length of the burrow was considered to be 100 cm since in previous studies the lengths of the burrows at the sampling sites near this study area were ranging between 80–100 cm [27].

### Data and statistical analysis

To compare the composition of sediment samples in the mud shrimp burrow and surroundings, Student’s t-test was applied to identify the differences between different sizes of sediment particles. The data for the proportion of sediment (%) and the proportion of AFDW in the sediment (%) were arcsine transformed in order to satisfy the assumptions of normality and homogeneity of variances. To identify the differences in the AFDW (carbon content) of the surroundings and mud shrimp burrow in three sampling areas, one-way analysis of variance (ANOVA) followed by post hoc Tukey’s honest significant difference (HSD) test were applied. To evaluate whether the carbon content inside the burrow was sufficient to support the shrimp living inside, the ratio of AFDW in MSBW and a single individual of shrimp (*A*. *edulis*) was calculated.

## Results

### Morphological traits of the mud shrimp burrow wall

**Fig 2:**
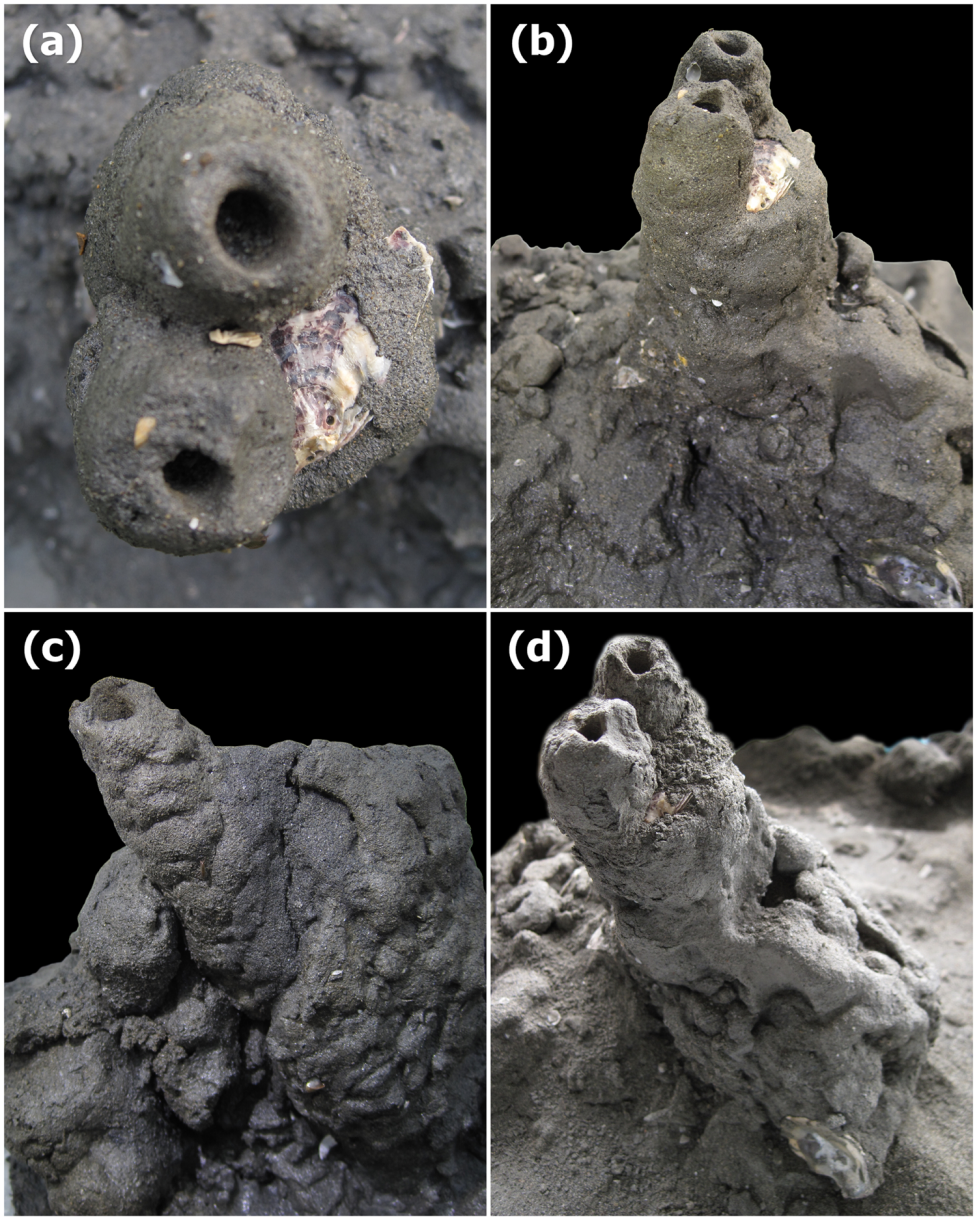
Photo of the mud shrimp burrow wall. Top view of the burrow showing the opening (a), the trunk of the burrow gradually thickening with depth (b), irregular deposition of the clay in the burrow wall (c), and intact morphology of the burrow wall 1 year after collection (d).

**Fig 3:**
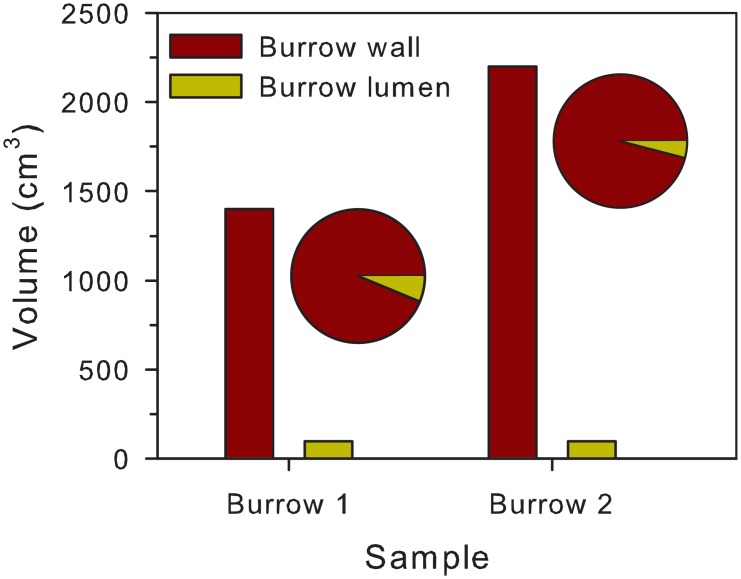
The volume of the burrow wall and the burrow lumen. Pie chart—the proportion of the burrow wall and the burrow lumen in a mud block.

The sediment texture of mud shrimp burrows appeared finer than the background sediment in all the three sampling areas. We studied the outer structure, which is the burrow wall, and the inner narrow tube, referred to as the burrow lumen from the portion of the mud block collected at 30cm depth. The burrow wall of the mud shrimp burrow was very broad and appeared huge in contrast to the burrow lumen, which only represented a thin narrow hollow tube of a confined shape (Fig 2). From the morphology of the burrow wall, a round-shaped opening was noted on the upper part (Fig 2a). Gradual thickening was significant from the top to the bottom of the burrow with the diameter being 4–5 cm at the top and 20–25 cm at the bottom (in about 30 cm depth). Distinctive portions are the opening of the burrow and a chimney-like trunk narrowing from the lower to the upper portion (Fig 2b). An extremely irregular deposition of thick mud without a distinct shape was noted on the outer surface of the burrow (Fig 2c). The trunk of the burrow appeared to be strong and not fragile when it got dry after 1 year (Fig 2d). The volume of the burrow wall showed a huge difference with the volume of the burrow lumen (Fig 3). The volume of the burrow wall was about 19.1 (± 6.0) times of the burrow lumen (n = 2).

### Composition of the mud shrimp burrow wall

**Fig 4:**
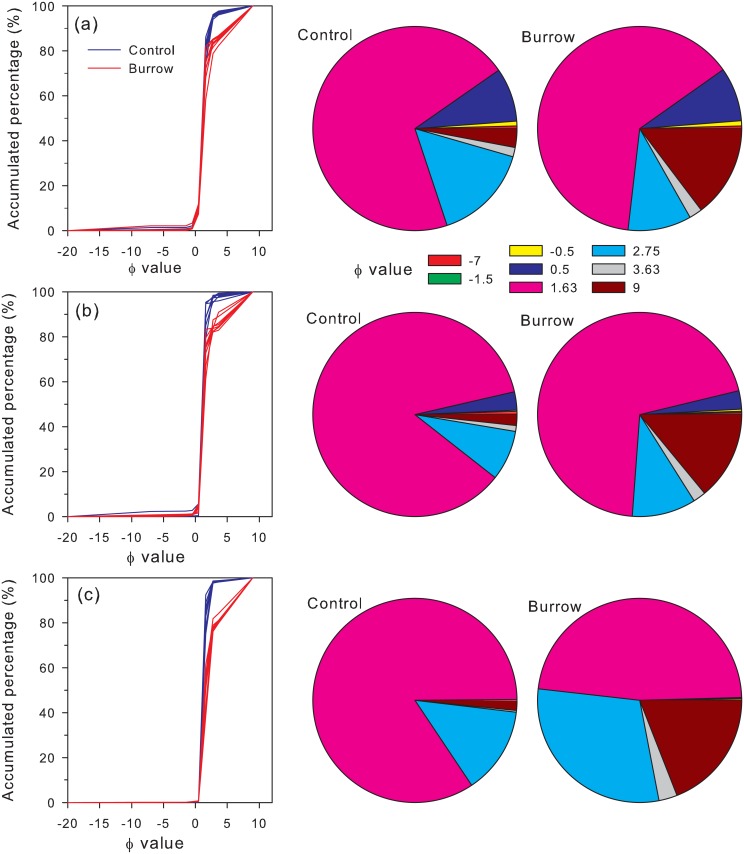
Accumulated percentage and proportion of sediments in burrow wall and surroundings from three sampling areas. Shengang (a), Hanbao (b), and Wangong (c).

The composition of MSBW showed a clear difference to background sediments in all three sampling areas (Fig 4). The composition of background sediments indicated that the mud shrimp used a habitat with a higher proportion of medium sand (> 70% of size Φ1.63) and clay (size Φ9). The accumulation of clay (size Φ9) in the burrow wall was noticed in all three study areas when compared with the background sediment (Fig 4). Most of the size categories in the MSBW were altered particularly in Wangong. When the results of all MSBW samples were combined, the Student’s t-test revealed that proportions of size Φ 1.63, were significantly higher in background samples than in burrow samples (t = 6.61, *p* < 0.001). Nevertheless, the proportions of size Φ -0.5 (t = -2.09, *p* = 0.043), 3.63 (t = -5.22, *p* < 0.001) and 9 (t = -25.01, *p* < 0.001) were significantly higher in burrow samples than in background samples (Table 2). Taken together, the results of the composition of MSBW revealed: (1) the ability and the preference of mud shrimps to select fine sediments to build their burrows, and (2) the changes in the sediment characteristics by the burrowing behavior of the mud shrimps is caused by a change of the physical sorting of sediment particles of the tidal flat by accumulating finer sediments inside their burrows.

**Table 2:** Results of Student’s t-test comparison for proportion (%) of each size category of sediment between habitat (control) and burrow.

Location	Shengang (n = 8)			Hanbao (n = 8)			Wangong (n = 8)			All (n = 24)		
Φ value	Control	Burrow	t-value,	Control	Burrow	t-value,	Control	Burrow	t-value,	Control	Burrow	t-value,
WSC			*p*-value			*p*-value			*p*-value			*p*-value
-7	0.32	0.32	0.70,	0.34	0.29	-0.27,	< 0.01	0.01	-0.47,	0.22	0.2	0.24,
Cobble			0.5			0.79			0.65			0.81
-1.5	0.08	0.08	-0.341,	0.13	0.2	-1.69,	0.02	0.02	-0.45,	0.08	0.1	-1.05,
Granule			0.74			0.11			0.66			0.3
-0.5	0.77	0.82	-0.82,	0.19	0.34	-2.60,	0.03	0.22	-8.85,	0.33	0.46	-2.09,
Very coarse sand			0.43			0.021*			0.001**			0.043*
0.5	8.51	8.55	-0.15,	2.9	2.9	-0.23,	0.08	0.19	-7.50,	3.83	3.88	-0.23,
Coarse sand			0.99			0.82			<0.001**			0.82
1.63	70.44	63.42	2.66,	85.82	70.13	4.77,	84.25	47.76	9.77,	80.17	60.44	6.61,
Medium sand			0.02*			<0.001**			<0.001**			<0.001**
2.75	15.42	10.04	2.42,	7.93	10.12	-0.59,	13.76	29.86	-4.89,	12.37	16.67	-1.26,
Fine sand			0.03*			0.57			<0.001**			0.21
3.63	1.48	2.08	-1.80,	0.93	2	-1.67,	0.27	2.92	-19.67,	0.9	2.33	-5.22,
Very fine sand			0.1			0.12			<0.001**			<0.001**
9	2.98	14.7	-24.61,	1.74	14.02	-12.82,	1.58	19.02	-45.22,	2.1	15.92	-25.01,
Clay			<0.001**			<0.001**			<0.001**			<0.001**

n is number of samples. WSC is Wentworth Size Class.

* Significant at the *p* < 0.05 level (2-tailed);

** significant at the *p* < 0.01 level (2-tailed).

**Fig 5:**
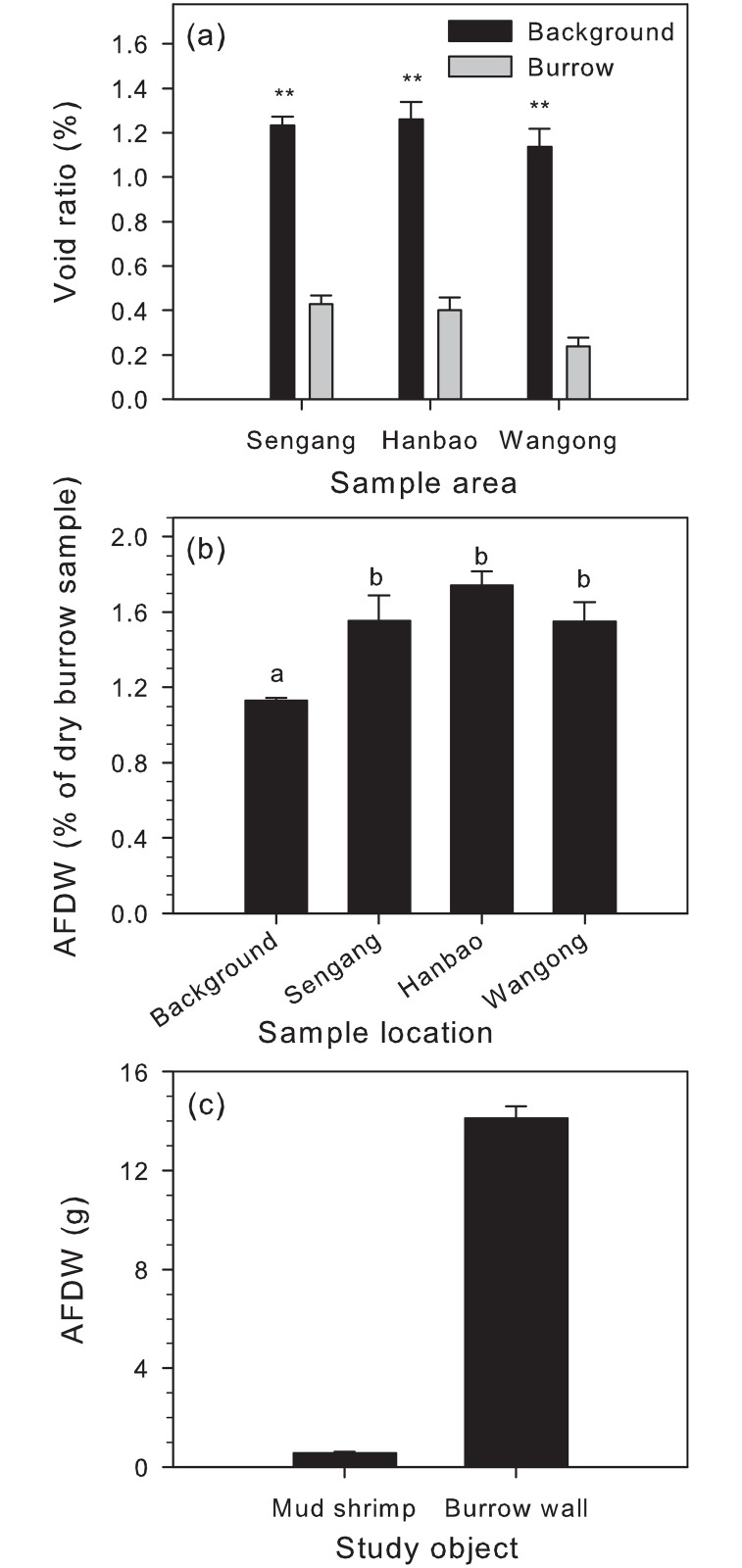
Comparison of the void ratio of the mud shrimp burrow wall and the ambient sediment in 3 sampling areas; (a) comparison of ash free dry weight of background sediment and mud shrimp burrow wall using one-way analysis of variance, followed by Tukey’s test; (b) relative weight of ash-free dry weight of one individual of mud shrimp and one whole mud shrimp burrow wall (c).

Furthermore, the traits of MSBW showed an average value of the void ratio (*e*): 0.43 ± 0.04 (%), 0.4 ± 0.06 (%) and 0.24 ± 0.04 (%) collected from Shengang, Hanbow and Wangong, respectively. The void ratio of the MSBW found in all three areas was very low. The results of Student’s t–test (d.f. = 6) indicated that void ratios were significantly higher in samples of the ambient environment than in mud shrimp burrows in Shengang (*p* < 0.001, t = -28.24), Hanbao (*p* < 0.001, t = -17.65), and Wangong (*p* < 0.001, t = -19.71). (Fig 5a)

### AFDW analysis

The content of AFDW in the three sampling areas varied, ranging between 1.55 ± 0.10 (% of dry burrow sample, Wangong) and 1.74 ± 0.08 (% of dry burrow sample, Hanbow). The statistical results showed no significant difference in MSBW among the 3 sampling areas, but all AFDW values in the MSBW were higher than the background (control) samples (*p* < 0.001, one-way ANOVA, Fig 5b). The average content of AFDW in MSBW was 1.23 ± 0.11 (% of dry burrow sample). By using the Eq (2) the AFDW in one entire burrow (burrow lumen 1cm thick) was found to be 14.17 ± 2.82 g.

The average AFDW of one adult mud shrimp was 0.586 ± 0.038 g, whereas no significant difference was found between the two sexes (*p* > 0.05, student T-test). Further, the AFDW in a single burrow was 24.2 times to the AFDW of one shrimp (Fig 5c).

## Discussion

### Burrow characteristics

The burrow of *A*. *edulis* comprises of an upper U-section and a central shaft, thereby giving an overall Y-shaped appearance [27]. The structure of the burrow of *A*. *edulis* is similar to suspension feeding upogebiid shrimps like having small and narrow circular tunnels [5, 19, 27,-28, 40]. The upper U-section of the burrow is generally formed for the exchange of water from outside to inside of the burrow since these shrimps are mainly filter feeders [27, 22, 41–42]. The burrow of *A*. *edulis* showed the presence of circular chambers, which is used for turning the body inside the burrow [27].

The burrow lumen was found to be a narrow tube with a definite dimension of arm width extending vertically into the sediment by building a central shaft [27]. Also, arm width, volume and the total depth of *A*. *edulis* burrows were significant positively correlated with size of the shrimp [27]. The present study is the first record showing the outer burrow wall of the *A*. *edulis* burrow. Several interspecific variations are known from Upogebiidae shrimp burrow morphology with respect to their structure, shape, and dimension [27, 42]. In the case of the deposit feeder ghost shrimp *Nihonotrypaea petalura*, the extension of the burrow was greater horizontally than vertically, having a single opening at the surface [43]. Even though the burrow lumen of *A*. *edulis* had a distinct Y-shaped appearance, the outer burrow wall was thick with an overall irregular shape and became extended with accumulated sediment. In a previous study, *Upogebia pusilla* was observed to push significant amounts of sediment against the burrow wall in order to considerably enlarge the burrow and build the burrow lining [19, 44]. The result of the present study supports the above inference about the behavior of the mud shrimp (*A*. *edulis*), because their burrowing behavior leads to a thick deposition of clayey particles that ultimately strengthen the burrow.

### Grain size distributions in burrow and background

Burrowing animals exhibit a strong influence on the physical characteristics of the sediment by altering the penetrability and permeability to water, and the water content of the burrow [37, 45, 46]. Studies on how the burrowing or other biological activities affect the physical characteristics of the sediment are few [47]. The pattern for the alteration of physical characteristics of the sediment for mud crab species like *Uca uruguayensis* and *Chasmagnathus granulata* was marked by higher penetrability and lower permeability [45]. Several studies reported the same phenomena about the effects of bioturbating animals [47, 48–50]. In the present study, *A*. *edulis* was found to accumulate finer sediments (clay) when building their burrow. This was also noticed in the crab *C*. *granulata* where the burrow is characterized by the accumulation of finer particles, which meant that they could selectively choose respective sediments for building their burrow wall [45]. Their report revealed the trapping of clayey or finer sediments inside the burrow tunnel during high tide. The present study also confirmed the presence of clay particles in the burrow wall when compared to the sediment composition of the background.

Some thalassinidean shrimps have funnel shaped burrow openings which are supposed to act as sediment traps by collecting clayey particles and accumulating them in their burrow linings [13, 22, 51]. The burrowing crabs are supposed to deposit the sediment in the form of mounds on the tidal flat over many tidal cycles, eventually covering the crab bed surface [45]. Furthermore, the accumulated sediments are cohesive, dense and not easy to transport [45]. This result was in accordance with the present study where the mud shrimp *A*. *edulis* accumulated clayey particles and deposited them in their burrow randomly, providing an overall irregular morphology of the burrow wall. The accumulation of fine sediments might also happen because of the breakdown of coarser sediments into finer particles by the mud shrimp while burrowing. Both possibilities reflect the alteration of the sediment characteristics by the mud shrimp, which provides a sedimentological impact on the mudflat [52].

The ability of selecting particles according to their size was noted before in thalassinidean shrimps in some studies [4, 53]. These studies were mainly associated with understanding the trophic modes of mud shrimps. For example, the trophic mode of *Upogebia omissa* was reported to have the ability to select finer particles based on size by re-suspension during deposit feeding [4, 53]. The present study showed that mud shrimps could separate particles during the burrowing process by separating finer sediments and accumulating them in their burrow.

In this study the void ratio in the burrow wall was found to be very small (less than 0.5%), and indicates a low permeability. A tendency of the shrimp to isolate themselves from the outer world by building strong, compact burrows with very small openings indicates that this animal does not need to access the surface [22]. A previous study on the mudflat amphipod *Corophium volutator* showed that the permeability of the sediment decreased with an increase in population density [47]. The results of these authors suggest an inverse relationship between shear strength and permeability of the sediment that might be responsible for biological activity. The result of the present study confirms that the influence of some mud flat animals can change certain characteristics of the sediment, which may, therefore, affect other benthic animals living in vicinity [54]. In fact, ecosystem engineers can create their own modified habitat by impacting the functioning and the structure of the ecosystem [55].

The low void ratio and the compactness due to the accumulation of clayey particles increase the shear strength of the burrow [47, 56, 57, 58]. These characteristics of the burrow can protect mud shrimps from predators [41]. Several studies noticed that upogebiid shrimps reduce the diameter of their burrow opening [19, 59]. This phenomenon of small openings could be to maximize the generation of currents and to hide from predators [22]. Some ghost shrimps could survive in very low oxygen and have their own response mechanisms in order to thrive under hypoxic conditions [60]. The present results support previous studies and highlight the fact that mud shrimps alter the physical characteristics of the sediment in order to build strong burrows.

### Organic carbon in the burrow wall

The content of the organic matter in the MSBW is an important parameter to calculate the available particulate organic matter as a trophic source for mud shrimps. Previous studies have shown higher values of organic matter in the burrow wall than those of the background sediment in most Upogebiidae shrimps [32, 36, 59–63]. In the present study, the organic content in the burrow wall was also found to be higher than in background sediments. In fact, a recent study reported high quality particulate organic matter (POM) as an essential component in the diet of the ghost shrimp *N*. *californiensis*. This POM reaches out to the bottom of the deep burrow commonly through sediment reworking or burrowing behavior of ghost shrimps [64].

From previous studies, the Upogebiidea have been reported mainly as filter feeding animals [22, 53, 65–68]. However, there are other studies that reflect different trophic modes and sometimes even more than a single mode [22, 68]. According to the report of Coelho et al. (2000), *U*. *omissa* had a strong tendency for deposit feeding; they described this species as a generalist feeder. This dual trophic behavior has been previously reported for *U*. *pusilla* [67] and *U*. *stellata* [22]. Hence, a detailed study of the burrowing and feeding behavior of mud shrimps is necessary.

In the present study, the value of AFDW of one entire mud shrimp burrow was 24 times higher than that of an adult mud shrimp. According to the ten percent rule in a trophic pyramid, during the transfer of energy from the organic food from a lower trophic level to a higher trophic level, approximately 10 percent of the energy from organic sources is transferred to the higher trophic level [69]. Shrimps belonging to the Upogebiidae are shown to have trophic plasticity [22]. A study on the callianassid shrimp *Callianassa subterranea* showed that ground dried algae and dried zooplankton can let these animals survive in the laboratory for more than 2 years [70, 71]. According to the report of Kinoshita et al. (2008), organic particles were easily trapped in the burrow of *Upogebia major*, which are mainly considered to be filter feeders. The present study gives an idea that the organic carbon content is significantly higher in the burrow than the background and this might be a possible source of trophic energy for the mud shrimp living inside.

## Conclusion

The mud shrimp *A*. *edulis* selects specific particle sizes during their burrowing activity and this way changes the sediment characteristics of mudflats. The ability to choose finer sediments for building their burrow was noticed. The structure of the burrow wall differed greatly from the burrow lumen. The burrow wall was thick with an accumulation of clayey particles. These clayey particles formed the burrow wall, which showed a low void ratio, thereby indicating a low permeability and higher sheer strength to protect the mud shrimp living inside the burrow. The burrow wall of *A*. *edulis* has almost 24 times higher organic content than one individual of mud shrimp. The shrimp might sustain its life with the available organic matter inside the burrow. These findings about the behavior of the mud shrimp *A*. *edulis* reflect a change or alteration of the mud flat characteristics with a probably substantial ecological impact. The particular mechanisms of fine sediment acquisition while building the burrow and the quantification of burrow strength of the mud shrimp *A*. *edulis* demand for in depth follow-up studies.

## Supporting information

S1 File Figure A1 in S1 File. The volume of the burrow wall and the burrow lumen. Pie chart—the proportion of the burrow wall and the burrow lumen in a mud block. Figure B1 in S1 File. Accumulated percentage and proportion of sediments in burrow wall and surroundings from three sampling areas. Shengang (a), Hanbao (b), and Wangong (c). Table B2 in S1 File.. Results of Student’s t-test comparison for proportion (%) of each size category of sediment between habitat (control) and burrow. n is number of samples. WSC is Wentworth Size Class. [* Significant at the *p* < 0.05 level (2-tailed); ** significant at the *p* < 0.01 level (2-tailed)]. Figure C1 in S1 File. Comparison of the void ratio of the mud shrimp burrow wall and the ambient sediment in 3 sampling areas. Figure C2 in S1 File. Comparison of ash free dry weight of background sediment and mud shrimp burrow wall using one-way analysis of variance, followed by Tukey’s test. Figure C3 in S1 File. Relative weight of ash-free dry weight of one individual of mud shrimp and one whole mud shrimp burrow wall.
(XLSX)
